# Supplementary material for: Deciphering Bitcoin Blockchain Data by Cohort Analysis
Source: Sci Data. 2022 Apr 7;9:136. doi: 10.1038/s41597-022-01254-0 (PMC8989952; doi:10.1038/s41597-022-01254-0)
Supplement: Supplementary file 1 — Appendix [file 41597_2022_1254_MOESM1_ESM.pdf]

Supplementary Material for "Deciphering Bitcoin Blockchain Data by Cohort Analysis"

Yulin Liu[1,4,†], Luyao Zhang[*,2,1,†], and Yinhong Zhao[3,1,†]

[*]Corresponding author: email: lz183@duke.edu, institution: Data Science Research Center and Social Science Division, address: Duke Kunshan University, No. 8 Duke Avenue, Kunshan, Jiangsu, 215316, China.
[1]SciEcon CIC, London, WC2H 9JQ, United Kingdom
[2]Data Science Research Center and Social Science Division, Duke Kunshan University, Kunshan, Jiangsu, 215316, China
[3]Duke University, Durham, NC, 27708, United States
[4]Bochsler Finance, Zug, 6300, Switzerland
[†]The authors are listed in alphabetical order according to last names and contributed equally to this work.

ABSTRACT

Bitcoin is a peer-to-peer electronic payment system that has rapidly grown in popularity in recent years. Usually, the complete history of Bitcoin blockchain data must be queried to acquire variables with economic meaning. This task has recently become increasingly difficult, as there are over 1.6 billion historical transactions on the Bitcoin blockchain. It is thus important to query Bitcoin transaction data in a way that is more efficient and provides economic insights. We apply cohort analysis that interprets Bitcoin blockchain data using methods developed for population data in the social sciences. Specifically, we query and process the Bitcoin transaction input and output data within each daily cohort. This enables us to create datasets and visualizations for some key Bitcoin transaction indicators, including the daily lifespan distributions of spent transaction output (STXO) and the daily age distributions of the cumulative unspent transaction output (UTXO). We provide a computationally feasible approach for characterizing Bitcoin transactions that paves the way for future economic studies of Bitcoin.

Supplementary Material

Meta Data

Table S1 and Table S2 present the meta data of the data we present on Bitcoin STXO and UTXO.

Table S3 lists all the data files published in our GitHub depository, including the STXO and UTXO data for six cryptocurrencies including Bitcoin.

Additional Visualizations

Figures S1, S2, S3, S4, S5, and S6 are several additional visualizations of the data we present here.

Name	Description
date	Date on which cohort data were queried, in the format "%Y/%m/%d"
newborn	Number of UTXOs in BTC created on the given date
dead	Number of UTXOs in BTC spent as inputs on the given date
WAL	Weighted average lifespan of the UTXOs spent on the given date, defined as the average lifespan (the difference between the time when the output was spent and the time when the output were created) weighted by the number of UTXOs in BTC contained in the transaction outputs.
-9	Number of UTXOs in BTC spent on the given date that were created less than one day (< 1d) before
-7	Number of UTXOs in BTC spent on the given date that were created more than one day but less than one month (1d $\sim$ 1m) before
-5	Number of UTXOs in BTC spent on the given date that were created more than one month but less than three months (1m $\sim$ 1q) before
-3	Number of UTXOs in BTC spent on the given date that were created more than three months but less than six months (1q $\sim$ 6m) before
-1	Number of UTXOs in BTC spent on the given date that were created more than six months but less than one year (6m $\sim$ 1y) before
1	Number of UTXOs in BTC spent on the given date that were created more than one year but less than two years (1y $\sim$ 2y) before
3	Number of UTXOs in BTC spent on the given date that were created more than two years but less than three years (2y $\sim$ 3y) before
5	Number of UTXOs in BTC spent on the given date that were created more than three years but less than four years (3y $\sim$ 4y) before
7	Number of UTXOs in BTC spent on the given date that were created more than four years but less than five years (4y $\sim$ 5y) before
9	Number of UTXOs in BTC spent on the given date that were created more than five years but less than ten years (5y $\sim$ 10y) before
11	Number of UTXOs in BTC spent on the given date that were created more than ten years (> 10y) before

Table S1. Meta Data for STXO Dataset

Name	Description
date	Date on which cohort data was queried, in the format "%Y/%m/%d"
-9	Number of UTXOs in BTC still alive by the end of the given date that were created less than one day (< 1d) before
-7	Number of UTXOs in BTC still alive by the end of the given date that were created more than one day but less than one month (1d $\sim$ 1m) before
-5	Number of UTXOs in BTC still alive by the end of the given date that were created more than one month but less than three months (1m $\sim$ 1q) before
-3	Number of UTXOs still alive by the end of the given date that were created more than three months but less than six months (1q $\sim$ 6m) before
-1	Number of UTXOs in BTC still alive by the end of the given date that were created more than six months but less than one year (6m $\sim$ 1y) before
1	Number of UTXOs in BTC still alive by the end of the given date that were created more than one year but less than two years (1y $\sim$ 2y) before
3	Number of UTXOs in BTC still alive by the end of the given date that were created more than two years but less than three years (2y $\sim$ 3y) before
5	Number of UTXOs in BTC still alive by the end of the given date that were created more than three years but less than four years (3y $\sim$ 4y) before
7	Number of UTXOs in BTC still alive by the end of the given date that were created more than four years but less than five years (4y $\sim$ 5y) before
9	Number of UTXOs in BTC still alive by the end of the given date that were created more than five years but less than ten years (5y $\sim$ 10y) before
11	Number of UTXOs in BTC still alive by the end of the given date that were created more than ten years (> 10y) before

Table S2. Meta Data for UTXO Dataset

name	brief	data end date	UTXO file name	STXO file name
Bitcoin	BTC	2021-02-10	BitcoinResultUTXO2021-02-10.csv	BitcoinResultSTXO2021-02-10.csv
Bitcoin Cash	BCH	2020-12-31	bitcoin_cashResultUTXO2020-12-31.csv	bitcoin_cashResultSTXO2020-12-31.csv
Litecoin	LTC	2020-12-31	litecoinResultUTXO2020-12-31.csv	litecoinResultSTXO2020-12-31.csv
Dogecoin	DOGE	2020-12-31	dogecoinResultUTXO2020-12-31.csv	dogecoinResultSTXO2020-12-31.csv
Dash	DASH	2021-02-20	dashResultUTXO2021-02-20.csv	dashResultSTXO2021-02-20.csv
Zcash	ZEC	2020-12-31	zcashResultUTXO2021-02-20.csv	zcashResultSTXO2021-02-20.csv

Table S3. File names of Altcoins UTXO and STXO data on GitHub

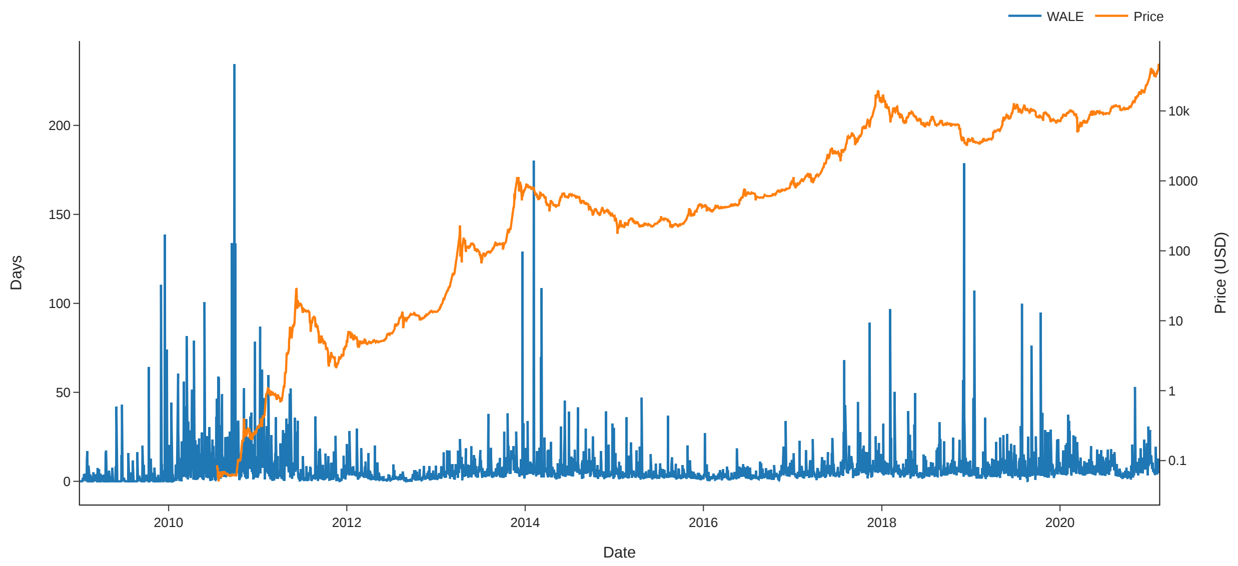

Figure S1. Daily weighted average lifespan of Bitcoin STXOs and BTC price. The figure shows that the WAL of BTCs in STXOs attains a peak value when the BTC price is volatile. For example, the 2014 peak of WAL value closely followed the rocketing of BTC price from $100 to $1000 and its subsequent price collapse. This implies that older BTC become more active during market turmoil.

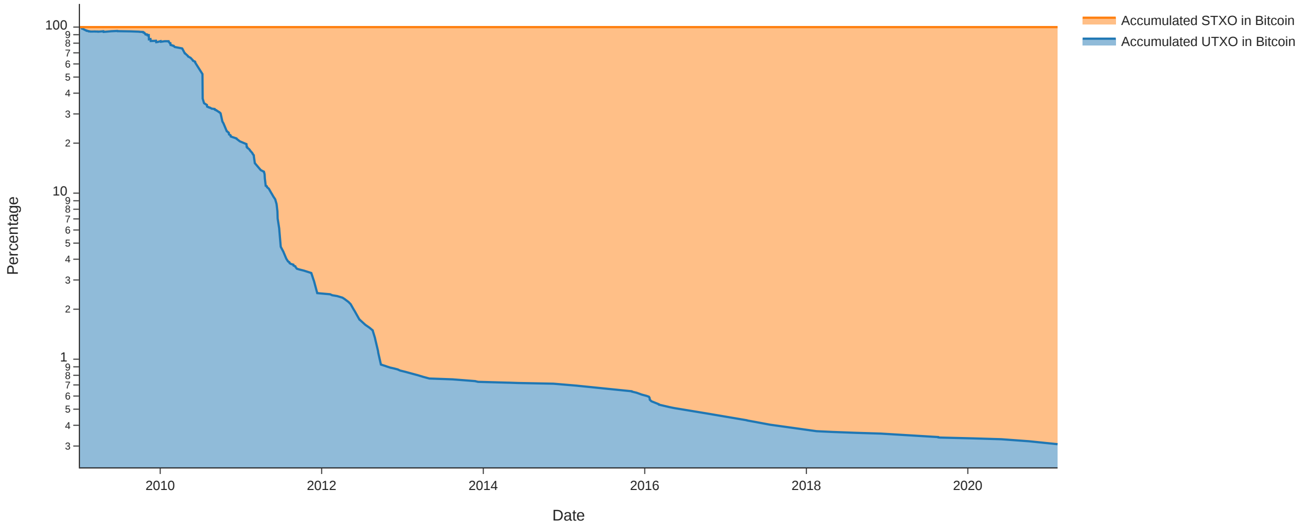

Figure S2. Percentage of STXO and UTXO on Bitcoin

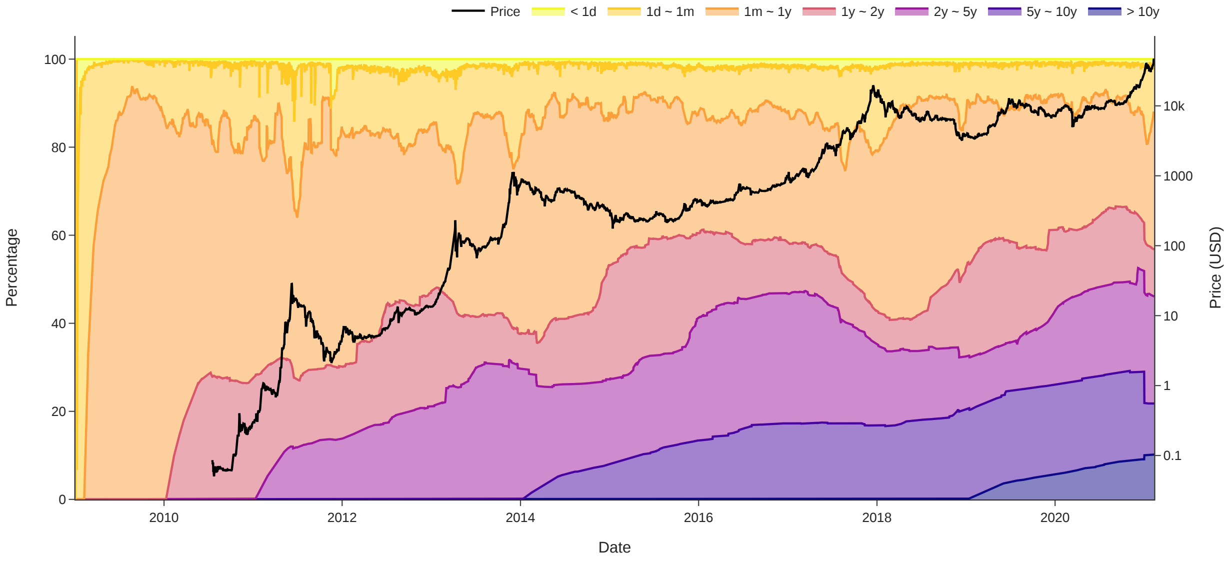

Figure S3. Age distribution of UTXO on Bitcoin and the price of BTC

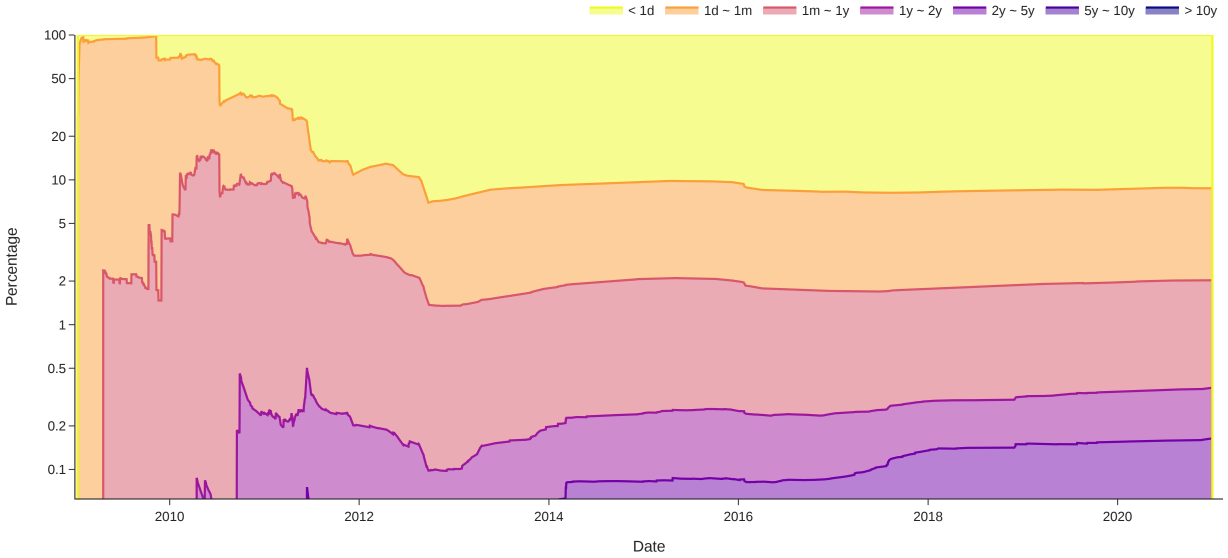

Figure S4. Cumulative lifespan distribution of STXO on Bitcoin

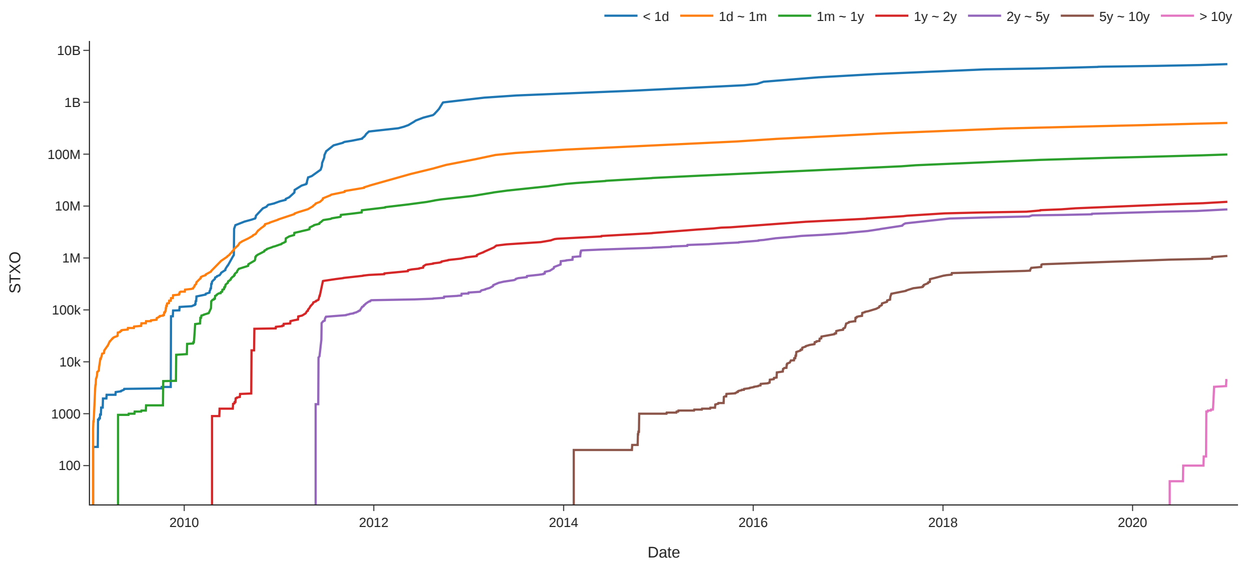

Figure S5. Cumulative STXOs by lifespan on Bitcoin

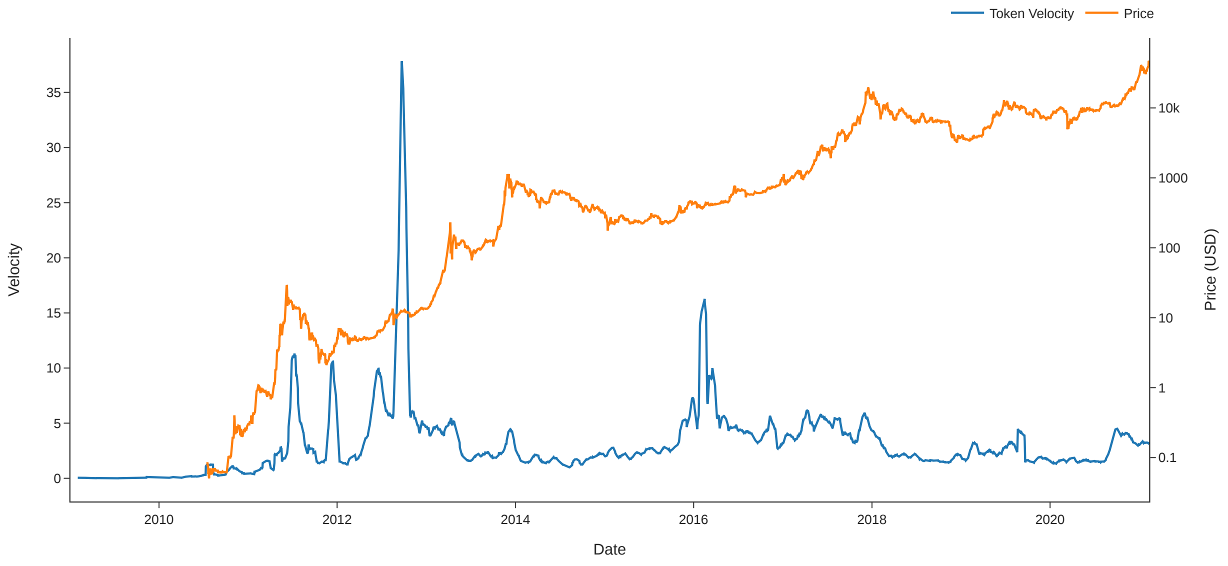

Figure S6. Token velocity and price of BTC
